# Potential of Chitosan-Based Systems Integrating Therapeutic Ions, Mesoporous Silica and SPIONs as Next-Generation Multifunctional Platforms for Diabetic Wound Healing

**DOI:** 10.3390/md24100347

**Published:** 2026-10-01

**Authors:** Radhika Radhika, Diana C. Lago, Zulema Vargas-Osorio

**Affiliations:** 1 Centre for Functional and Surface Functionalized Glass (FunGlass), Alexander Dubček University of Trenčín, 911 50 Trencín, Slovakia; radhika.radhika@tnuni.sk (R.R.); diana.lago@tnuni.sk (D.C.L.); 2 Nanotechnology and Magnetism Group, NANOMAG Laboratory, Applied Physics Department, iMATUS Materials Institute, Universidade de Santiago de Compostela, 15782 Santiago de Compostela, Spain

**Keywords:** diabetic wounds, mesoporous silica, multifunctional chitosan-based systems, next-generation platforms with therapeutic and diagnostic capabilities, SPIONs, therapeutic ions

## Abstract

Diabetes impairs multiple stages of the wound-healing process, resulting in chronic, infection-prone wounds that pose a substantial clinical burden. Despite available therapeutic options, effective management remains challenging owing to inadequate treatment efficacy, increasing antimicrobial resistance, and potential adverse side effects. Diabetic wounds exhibit a complex pathological microenvironment characterized by persistent bacterial colonization, hyperglycemia, tissue hypoxia, chronic inflammation, excessive matrix metalloproteinase activity, oxidative stress, and pH dysregulation. Nonetheless, traditional treatment strategies continue to rely largely on passive protection, inadequately addressing the underlying biological mechanisms responsible for impaired healing. Chitosan (CS), a naturally occurring cationic polysaccharide with high biocompatibility, antimicrobial activity, hemostatic properties, and a chemically tuneable structure, represents an attractive platform for advanced wound dressings. This review explores the potential of engineered chitosan-based systems for diabetic wound management through the strategic integration of therapeutic ions (Cu^2+^, Zn^2+^, Mg^2+^, Ag^+^, Mn^2+^), mesoporous silica nanocarriers, and superparamagnetic iron oxide nanoparticles (SPIONs). Unlike previous reviews that have primarily examined these components individually, the present work provides the first comprehensive analysis of their integration within a single multifunctional chitosan-based platform. By systematically evaluating their complementary therapeutic and diagnostic functions, including antimicrobial activity, angiogenic stimulation, oxidative stress modulation, controlled therapeutic delivery, and magnetic field-responsive behavior, this review establishes a unified design framework for the development of next-generation chronic wound dressings. Furthermore, it identifies potential synergistic interactions, key translational challenges, and future research directions required for the development of clinically relevant smart systems. Collectively, this integrated strategy offers a promising approach to overcoming the complex biological barriers that hinder diabetic wound healing.

## 1. Introduction

Diabetes is one of the most prevalent chronic diseases worldwide, affecting approximately one in nine adults. According to the International Diabetes Federation (IDF), the global prevalence of diabetes reached 11.1% in 2024, corresponding to nearly 589 million adults aged between 20 and 79 years. This number is projected to increase to 853 million by 2050, driven by factors such as urbanization, sedentary lifestyles, rising rates of overweight and obesity, and population ageing [1]. Diabetes is a widespread metabolic disorder characterized by chronic hyperglycemia resulting from impaired insulin secretion, insulin action, or both. Sustained elevated glucose levels contribute to dysfunction of multiple organs and systems, including the peripheral nerves, retina, kidneys, cardiovascular system, and blood vessels [2]. Among its most debilitating complications is diabetic peripheral neuropathy, which impairs sensory perception, compromises protective responses to injury, and significantly contributes to the onset and persistence of diabetic wounds [3]. Wound healing in diabetic patients is further hindered by complex pathological conditions, accompanied by a range of symptoms resulting from hyperglycemia. Consequently, diabetic wounds often fail to progress through the normal healing cascade, leading to chronic non-healing wounds, tissue necrosis, and severe infections that substantially increase the risk of amputation and mortality. Beyond their clinical impact, diabetic wounds impose a considerable socioeconomic burden on patients and healthcare systems worldwide, representing a major public health challenge [4].

### 1.1. Wound Healing Process

From a pathophysiological perspective, wound healing is a meticulous process consisting of four sequential stages: hemostasis, inflammation, proliferation and remodeling [5]. Hemostasis involves rapid clot formation to prevent blood loss [6], followed by an inflammatory response that recruits immune cells and releases cytokines and growth factors essential for tissue repair [7]. During the proliferative phase, fibroblast proliferation, angiogenesis, granulation tissue formation, and extracellular matrix deposition contribute to wound closure, whereas the remodeling phase involves collagen maturation and tissue reorganization to restore structural [8,9]. When the normal wound healing process is disrupted, wound closure is delayed, potentially leading to chronic wounds. The prolonged healing time of diabetic wounds, often extending over several months to years and sometimes not healing fully due to complex microenvironment. In diabetes, wound healing is profoundly impaired by a complex pathological microenvironment characterized by persistent hyperglycemia, chronic inflammation, oxidative stress, tissue hypoxia, impaired angiogenesis, neuropathy, and bacterial colonization [10]. Hyperglycemia promotes the formation of advanced glycation end products (AGEs), which alter cellular signaling, impair fibroblast and keratinocyte function, and reduce collagen synthesis. Simultaneously, excessive reactive oxygen species (ROS) production exacerbates oxidative stress, resulting in cellular damage and prolonged inflammation [11]. Furthermore, vascular dysfunction and hemoglobin glycation compromise oxygen delivery to injured tissues, creating a hypoxic environment that impairs cell proliferation, angiogenesis, and granulation tissue formation. Diabetic wounds typically exhibit sustained infiltration of neutrophils and pro-inflammatory macrophages, leading to elevated levels of inflammatory cytokines and proteases, particularly matrix metalloproteinases (MMPs), which accelerate extracellular matrix (ECM) degradation and hinder tissue regeneration. The expression and activity of key growth factors involved in tissue repair, including VEGF, PDGF, TGF-β, FGF, EGF, and IGF-1, are also markedly reduced, further delaying wound closure [12]. In addition, diabetic neuropathy diminishes protective sensation and alters neurogenic signaling, increasing susceptibility to repeated injury and infection. These interconnected abnormalities establish a self-perpetuating cycle of inflammation, tissue destruction, and impaired regeneration, making diabetic wounds particularly difficult to heal and highly prone to recurrence [13]. Multiple factors contribute to impaired wound healing in diabetes, as summarized in Figure 1. Consequently, the development of more effective therapeutic strategies for diabetic wound management has become a major challenge for both the medical and scientific communities, highlighting the urgent need for advanced biomaterials capable of addressing the complex pathological environment associated with chronic diabetic wounds [14].

**Figure 1:**
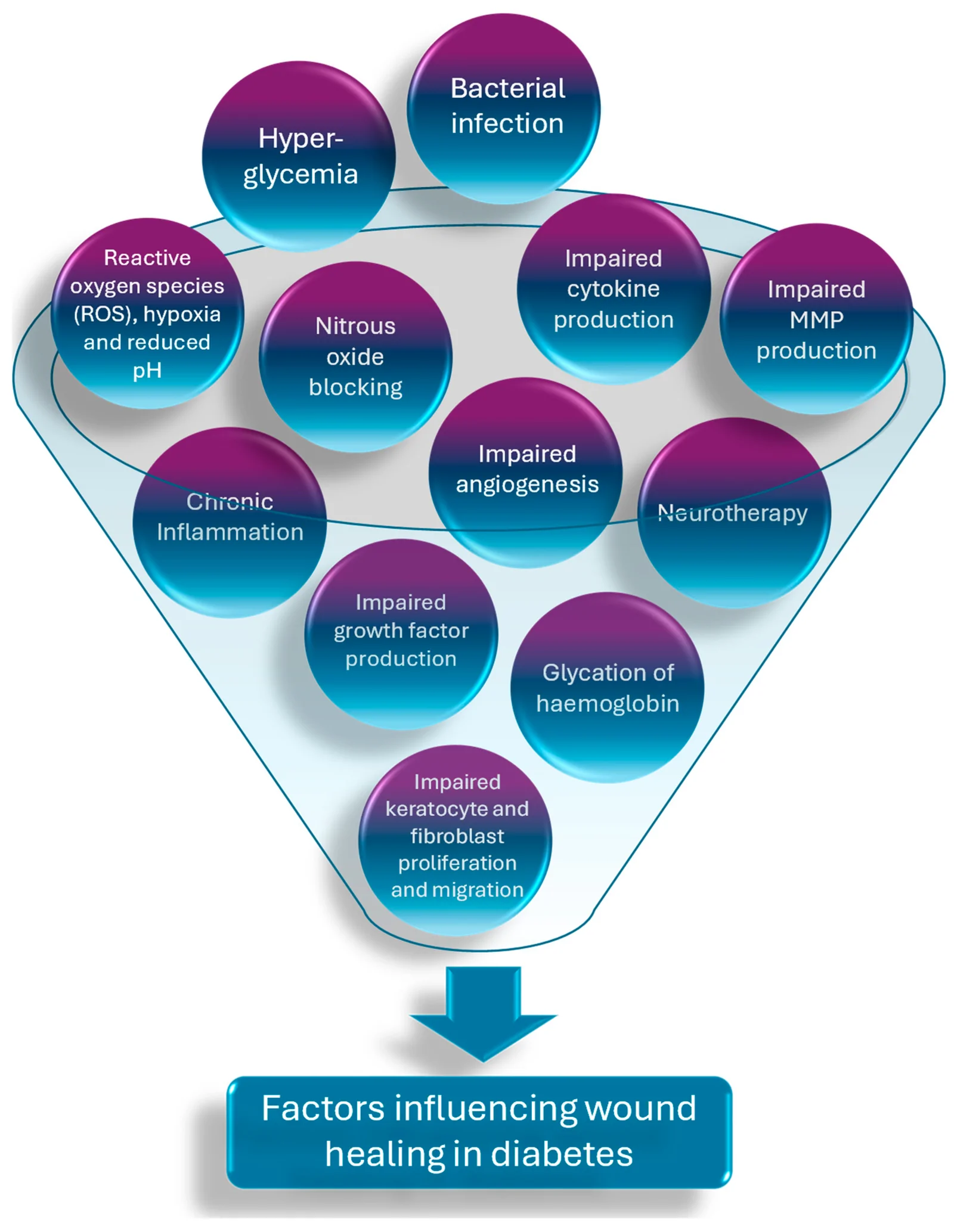
Factors affecting wound healing in diabetes.

### 1.2. Strategies to Treat Diabetic Wound Healing

Conventional therapeutic approaches for diabetic wound management often suffer from limitations such as poor retention at the wound site, limited tissue penetration, prolonged healing times, and insufficient support for tissue regeneration. Current clinical interventions, including wound dressings, debridement, off-loading, revascularization procedures, antibiotic therapy, and negative-pressure wound therapy, remain widely used but are frequently associated with high treatment costs and variable clinical outcomes, particularly in chronic and complex wounds [15]. Table 1 summarizes the conventional therapies currently used for diabetic wound healing. Notably, pivotal clinical trials evaluating novel therapies approved by the U.S. Food and Drug Administration (FDA) have reported healing rates of only 20–50% after 12 weeks. To address the multifactorial nature of diabetic wound healing impairment, a wide range of therapeutic strategies has been developed [16]. Nevertheless, traditional wound dressings provide limited biochemical stimulation and are generally unable to achieve precise and sustained delivery of therapeutic agents, reducing their regenerative potential [17]. Consequently, advanced biomaterials have gained considerable attention as multifunctional delivery platforms capable of transporting bioactive molecules, such as genes, peptides, proteins, mRNA, and growth factors, directly to the wound site, thereby promoting tissue regeneration and improving therapeutic efficacy [18].

**Table 1:** Conventional treatment used for diabetic wound healing: mechanisms, examples, advantages, and limitations.

Therapies	Description	Example	Advantages	Limitations	Ref
Wound debridement	Removal of necrotic or infected tissue from the diabetic wound	Surgical, enzymatic, mechanical and ultrasound debridement	Accelerate granulation and reduces risk of infection	Painful may require anesthesia, high risk of bleeding and repeated sessions required	[19]
Off-loading pressure	Reduce pressure on the diabetic wound site	Specialized diabetic footwear, crutches	Promotes diabetic wound healing	Expensive, poor compliance, may require lifestyle changes	[20]
Negative pressure wound therapy (NPWT)	Application of suction to remove exudate	Vacuum-assisted closure devices	Promotes wound healing and reduces bacterial burden	High-cost and require specialized equipment	[21]
Hyperbaric oxygen therapy chamber	Breathing pure oxygen under high pressure to increase oxygen delivery to wound site	Oxygen therapy chamber	Increases oxygen delivery, useful in hypoxic wounds	Expensive, time-consuming, risk of oxygen toxicity and the possibility of lung collapse	[22]
Platelet-rich plasma therapy	Injections or gels of platelet-rich plasma	Platelet-rich plasma (PRP) gels	Enhance tissue repair and reduce inflammation	Require multiple sessions	[23]
Stem cell therapy	Injection of stem cells into the wound	Bone marrow MSCs and adipose-derived stem cells	Stimulate tissue growth	Ethical concerns, expensive and require multiple sessions	[24]
Antibiotics	Prescription of antibiotics to reduce bacterial contamination on wound surface	Clindamycin, sulfamethoxazole and trimethoprim	Prevents infection	Antibiotic resistance, allergic reactions	[25]
Wound dressings	Maintain a moist environment and promote healing	Hydrogel, hydrocolloid dressings and gauze	Protect wounds, absorb exudate, prevent infection and promote healing	May require frequent changes and monitoring	[26,27]

Management of diabetic wound complications requires a personalized approach based on the patient’s clinical condition and wound severity. Accordingly, treatment strategies are typically tailored following a comprehensive clinical evaluation [28]. However, the multifactorial pathophysiology of diabetic wounds, characterized by persistent inflammation, oxidative stress, infection, and impaired tissue regeneration, together with the limitations of current therapies, underscores the need for multifunctional wound dressings capable of actively modulating the wound microenvironment [29]. These challenges have stimulated the development of advanced therapeutic platforms that extend beyond passive wound protection to promote targeted and sustained tissue repair.

## 2. Emerging Multifunctional Platforms for Diabetic Wound Healing

Wound dressings play a crucial role in tissue repair by maintaining a favorable microenvironment that supports the healing process [30]. An ideal wound dressing should combine biocompatibility, biodegradability, anti-inflammatory and antimicrobial properties, effective exudate management, moisture retention, and the ability to prevent secondary infections while promoting angiogenesis and tissue regeneration [31]. These systems can be fabricated from natural, modified, or synthetic polymers, either individually or in combination. Among them, biopolymers have attracted considerable attention owing to their high-water retention capacity, high biocompatibility, and ability to mimic the extracellular matrix (ECM), thereby providing a supportive environment for tissue regeneration and wound healing [32]. Polysaccharides are among the most abundant naturally occurring biopolymers and are composed of either homogeneous or heterogeneous assemblies of monosaccharide units. Variations in monosaccharide composition, glycosidic linkages, and macromolecular architecture confer a wide range of physicochemical and mechanical properties, including biodegradability, hydrophilicity, and structural versatility. These characteristics have enabled their extensive use in tissue engineering, drug delivery, and wound-healing applications [33]. Furthermore, the abundance of reactive functional groups facilitates chemical modification and the incorporation of therapeutic or stimuli-responsive moieties, expanding their functional potential in biomedical applications [34]. Polysaccharides can be obtained from plants, animals, microorganisms, and marine sources, with alginate, cellulose, chitosan, carrageenan, dextran, and hyaluronic acid representing some of the most widely investigated examples [35]. Despite the considerable potential of polysaccharide-based dressings, the complex and multifactorial pathophysiology of diabetic wounds demands therapeutic solutions that extend beyond passive wound coverage. Among these materials, chitosan has emerged as one of the most promising candidates for next-generation wound care owing to its intrinsic biocompatibility, biodegradability, hemostatic activity, and tunable chemical structure. These properties enable the development of multifunctional platforms capable of actively modulating the wound microenvironment and supporting multiple stages of the healing process [36]. Importantly, the strategic incorporation of therapeutic ions (such as Cu^2+^, Zn^2+^, Mg^2+^, and Ag^+^), mesoporous silica materials, and superparamagnetic iron oxide nanoparticles (SPIONs) within chitosan-based matrices introduces a new paradigm in wound dressing design. Therapeutic ions contribute antimicrobial, pro-angiogenic, and immunomodulatory functions [37], while mesoporous silica materials provide high surface area and tunable porosity for the efficient loading and sustained delivery of bioactive agents [38]. In parallel, SPIONs enable magnetically responsive functionalities, including targeted therapy, hyperthermia-mediated antibacterial effects, and diagnostic imaging capabilities [39]. The synergistic combination of these components with chitosan creates multifunctional platforms capable of simultaneously addressing infection, impaired vascularization, oxidative stress, and deficient tissue regeneration, thereby advancing wound care from passive coverage toward intelligent, responsive, and therapeutic systems with diagnosis functionalities for diabetic wound management.

The following sections systematically examine the potential of these components for diabetic wound-healing applications. Furthermore, this review emphasizes their synergistic integration within a single multifunctional platform. In particular, Section 7 consolidates current evidence by analysing representative studies conducted by Vargas-Osorio, et al., which report multifunctional systems with structural and compositional features analogous to those proposed herein. These investigations provide valuable insights into the interactions among the different components and establish a strong scientific rationale supporting the feasibility and potential therapeutic benefits of the proposed platform.

Figure 2 illustrates the synergistic integration of therapeutic ions, mesoporous silica materials, and SPIONs (magnetite nanoparticles) within a chitosan-based dressing. In this engineered platform, each component contributes distinct therapeutic and diagnostic functionalities while acting co-operatively to enhance the overall performance of the system. The chitosan matrix serves as the structural framework, where therapeutic ions are generally complexed or electrostatically attached to its amino or hydroxyl groups. Mesoporous silica materials are embedded within the polymer network, providing high-surface-area reservoirs for the loading and delivery of therapeutic agents. SPIONs may be directly dispersed throughout the matrix or immobilized onto mesoporous silica carriers prior to incorporation. The underlying architecture, its inherent design flexibility, and the specific rationale for combining these four components collectively underpin the multifunctional wound dressing concept presented in this review.

**Figure 2:**
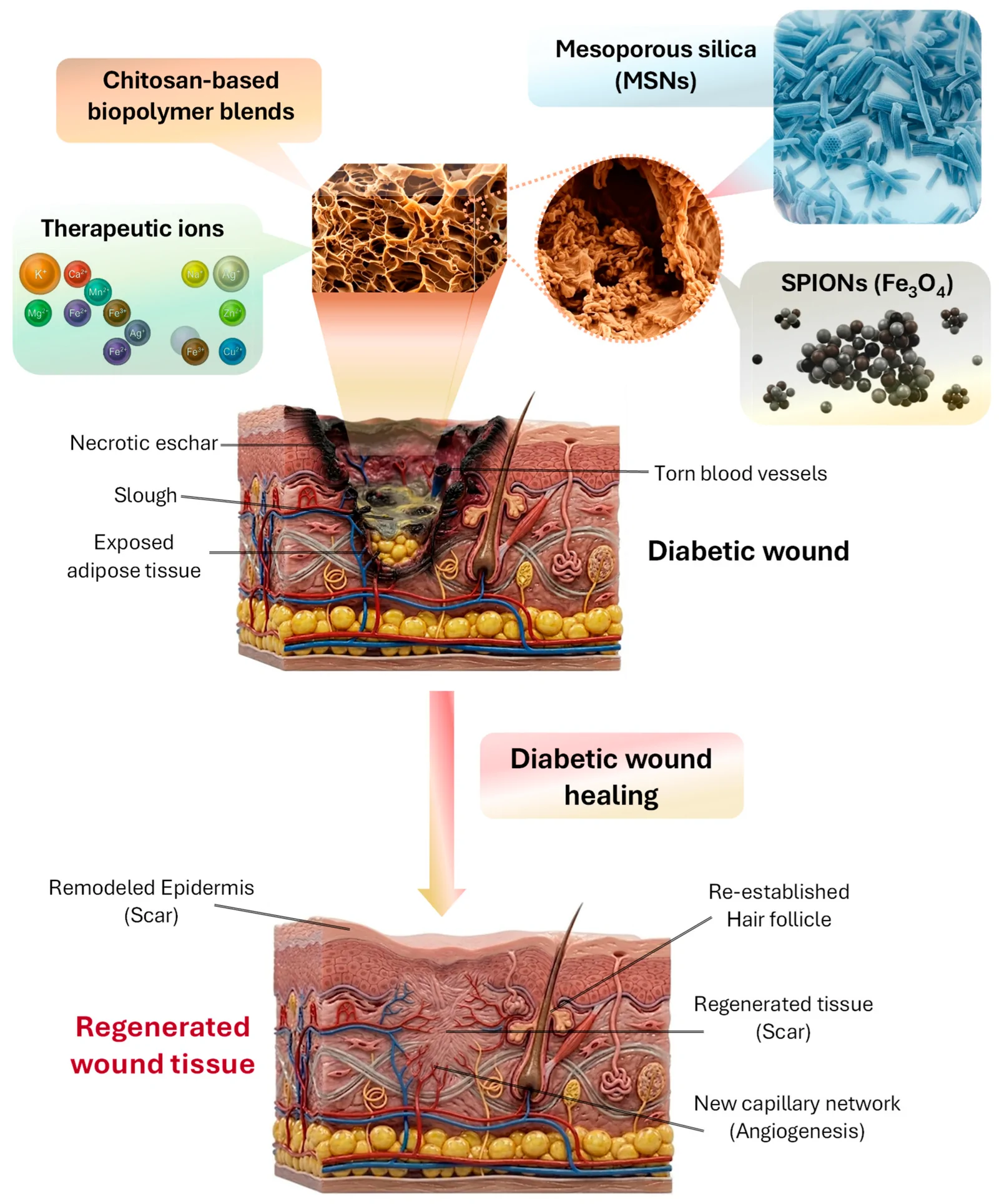
Next generation multifunctional systems with therapeutic and diagnostic capabilities for diabetic wound healing. Parts of this figure were created with the assistance of artificial intelligence (AI) tools and were subsequently reviewed and edited by the authors.

## 3. Chitosan—A Versatile Biopolymer in Biomedical Application

Chitosan, a naturally sourced polysaccharide, stands out among the various biomaterials investigated for wound dressing [40]. It is recognized as the second most abundant polysaccharide on Earth and is found in nature as structured microfibrils within the exoskeleton of mollusks and crustaceans, as well as in the fungi and insect cuticles [41]. Chitosan is a linear polysaccharide obtained from chitin through deacetylation, consisting of repeating β-(1-4)-linked N-acetyl-glucosamine units. The degree of deacetylation (DD) plays a crucial role in determining the balance between free amino groups (-NH2) and acetylated groups (-OAc), which directly impacts its properties [42]. This distinctive structure imparts several significant biological and physicochemical properties. The amino and hydroxyl functional groups facilitate strong hydrogen bonding, which is vital for water solubility and gel-formation. These groups also promote bioadhesion, enabling effective interactions with biological surfaces [43]. Additionally, amino groups serve as nucleophiles, allowing the binding of negatively charged molecules, such as growth factors or therapeutic agents, making chitosan suitable for controlled drug delivery applications. Conversely, chitosan with a higher crystallinity exhibits greater rigidity and mechanical strength, making it particularly advantageous for tissue engineering applications. In addition, the molecular weight and degree of polymerization of chitosan significantly influence its mechanical properties, biodegradability and bioavailability. Lower-molecular-weight chitosan generally exhibits enhanced water solubility and bioavailability, whereas higher-molecular-weight chitosan provides greater gel strength, viscoelasticity, and structural stability [44].

### Versatility of Chitosan for Its Use in Diabetic Wound Healing

The structural unit of chitosan contains numerous functional groups, including amino group, acetylamino, and primary hydroxyl groups, which considerably facilitates its chemical modification to produce various derivatives with distinct application properties. Chitosan is characterized by its antioxidant properties [45], low-toxicity, biocompatibility, antibacterial activity [46], biodegradability [47], and hemostasis abilities [48], all of which contribute positively to wound healing [49]. Its chemical structure can be adjusted to endow it with stimuli-responsive, injectable, and self-healing capabilities, thus addressing the limitations of traditional dressings that serve only a single function at a specific wound healing stage. This opens new avenues for the development of multifunctional dressings for diabetic wound treatment [50]. These advancements aim to improve delivery at the wound site, promote tissue regeneration, and dynamically adapt to local wound environments. Beyond its native form, chitosan can be chemically modified to obtain a wide range of derivatives, including carboxymethyl [51], phosphorylated [52], glycol [53], thiolated [54] and trimethyl chitosan [55]. The free amino group in the glucosamine unit of chitosan is typically targeted for chemical modification [56]. The versatility of chitosan is further evidenced by its compatibility with a variety of crosslinking strategies, including the use of 1,4-butanediol diglycidyl ether (BDDE), a crosslinking agent approved by FDA for applications involving hyaluronic acid. The incorporation of BDDE can significantly enhance the mechanical stability, structural integrity, and degradation resistance of chitosan-based fibrous systems, thereby extending their functional lifespan under physiological conditions and broadening their applicability in regenerative medicine and wound-healing applications [57]. Furthermore, chitosan can be combined with various polymers—particularly those with negative charges—to enhance its electrostatic properties, and modified through complexation strategies to incorporate a wide range of therapeutic ions. These modifications also improve its solubility at physiological pH, stability, and mucoadhesion, in addition to expanding its antimicrobial and tissue-regenerative functionalities [58,59,60]. Its inherent adaptability, along with its low immunogenicity, establishes chitosan as a leading platform capable of addressing a broad spectrum of medical challenges. Recent advances in biomaterials research have further highlighted its potential for diabetic wound management by enabling the development of multifunctional systems that simultaneously promote tissue repair, control infection, maintain a favorable wound environment, and modulate chronic inflammation. Consequently, chitosan-based materials represent a promising therapeutic strategy for chronic diabetic wounds, as summarized in Table 2.

**Table 2:** Summary of various chitosan-based platforms used for the treatment of diabetic wound healing.

Chitosan-Based Platform	Composition/Functionalization	Bioactive Agent	Major Outcomes Reported	Ref
Thermo-sensitive composite hydrogel	Chitosan and poloxamer (P407/P188)	Berberine and resveratrol	No hepato/nephrotoxicity, enhanced collagen deposition and regulating inflammatory factors. Exhibited more inhibitory effects on gram-positive bacteria than gram-negative bacteria	[61]
Injectable hydrogel	Carboxymethyl chitosan modified via methacrylic anhydride and phenylboronic acid	Quercetin and copper self-assembled nanoparticles	Suitable for irregular anatomical wound shapes, exhibits photo- ROS- and glucose responsivity. Promotes angiogenesis and regulating immunomodulatory	[62]
Microneedle patch	Ternary polymerizable deep eutectic solvent (PDES) system with a recyclable eutectogel backing layer and chitosan-based glucose-responsive nanoparticles	Insulin ad curcumin	Facilitated glucose-responsive insulin release, reducing blood glucose to 18.7% of initial level within 3 h. Acceleration healing with reduced inflammation and achieved 68.08% wound closure by day 10.	[63]
Glucose-responsive hydrogel	Phenylboronic acid functionalized chitosan and dopamine grafted gelatin	Dihydromyricetin and copper nanoparticles	Provides anti-inflammatory effects and antibacterial action against *S. aureus*. Significantly heal wound in 14 days.	[64]
pH-sensitive hydrogel	Carboxymethyl chitosan, oligoprocyanidins and oxide dextran	Deferoxamine	pH -triggered drug release and antioxidant. Up-regulating anti-inflammatory factors (IL-4 and IL-10) and promotes angiogenesis-related factors (HIF-1α, VEGF, CD31)	[65]
Biomimetic composite scaffold	Chitosan, oxidized isabgol (Oisab)	L- Arginine	Epithelialization—21.67 ± 1.6 days which was faster than control. Reduced CD68 + macrophages confirming early inflammation and robust angiogenesis. Improved collagen deposition and promoting neovascularization	[66]
Hydrogel dressing	Catechol-functionalized methacryloyl chitosan (CFMC) + polyacrylic acid	Vanillin and Silver nanoparticles (AgNPs)	Bacterial inhibition >89% against *S. aureus* and >90% against *E. coli*, significant ROS reduction, >95% infected wound closed by day 14, biodegraded in 21 days, no systematic toxicity	[67]
Electro-spun nanofibers membrane	Polyvinyl alcohol and chitosan	Cerium oxide nanoparticles (CeNPs) and mupirocin (MP)	Intracellular ROS reduction, DPPH scavenging, complete MSSA/MRSA elimination within 24 h, enhanced angiogenesis and skin regeneration	[68]
Multicomposite film	Chitosan film integrated with pH-sensitive purple cabbage anthocyanins	Copper sulfide nanoparticles (CuS NPs), gentamicin (Gent), purple cabbage anthocyanins (PCA)	Provides real-time visual infection monitoring via pH- responsive color changes. Exhibiting superb antibacterial properties 95.73% through synergistic photothermal therapy and antibiotic release.	[69]
Electrical stimulated gauze dressing	Chitosan and vaseline/paraffin oil (2:1)	Endogenous electric field mimic via HVMPC	No electrical burns with surface electrode. Accelerated wound healing from day 3, regulating growth factors in wound tissue by day 7 and inhibiting scar formation	[70]

As summarized in Table 2, stimulus-responsive hydrogels represent the majority of reported chitosan-based platforms, followed by microneedle patches, films, scaffolds, and fibrous/gauze systems. Despite significant progress, most reported chitosan-based platforms comprise only two, and occasionally three, functional components. Furthermore, meaningful comparisons between studies are often precluded by variations in experimental models, material compositions, and testing conditions, limiting the ability to accurately evaluate their relative performance and therapeutic benefits. Advances in nanotechnology have facilitated the incorporation of therapeutic metal ions into chitosan-based formulations, providing a promising strategy to enhance antibacterial activity, promote angiogenesis, and support cellular proliferation and differentiation. By leveraging the complementary properties of chitosan and bioactive metal ions, these multifunctional systems can accelerate tissue regeneration, reduce the risk of infection, and improve overall wound-healing outcomes [71].

## 4. Therapeutic Ions for Enhanced Wound Healing in Diabetes

Inorganic components mainly comprise nanoparticles based on therapeutic metals and metal oxides. In addition, the ability of metal ions to form stable coordination complexes with drug molecules provides an effective strategy for enhancing binding and sustained drug release at wound sites, making them highly useful in formulations intended for diabetic wound healing. The metal ions frequently used for this purpose include copper, zinc, magnesium, silver and manganese. These therapeutic metal nanoparticles are highly effective for sterilization, employing mechanisms such as interaction with bacterial cell walls to cause degradation or inhibiting DNA replications [32]. Among the therapeutic ions examined in this review, the biologically significant oxidation state is mainly divalent (^2+^), such as Cu^2+^, Zn^2+^, Mg^2+^, and Mn^2+^. Silver is an exception, as its wound-healing properties are linked to its monovalent Ag^+^ state. Other oxidation states of these metals are less common in physiological contexts and are typically linked to different, often non-therapeutic applications (such as industrial catalysis or redox chemistry) rather than wound healing.

### 4.1. Copper

Copper is an essential element for humans and other mammals and plays a significant role in numerous essential biological pathways [72]. Copper nanoparticles (CuNPs) are biocompatible and can be fabricated using environmentally friendly techniques [73]. Both copper ions and CuNPs are redox-active species [74]. Copper is crucial for various physiological functions, including all four stages of wound healing. Cu^2+^ can promote angiogenesis and enhance the expression of skin-related proteins in diabetic wounds. The proangiogenic properties of copper are attributed to the increased expression and activation of angiogenesis related factors, such as VEGF, fibroblast growth factor 1 (FGF1), platelet-derived growth factor (PDGF), interleukin—8 (IL-8), interleukin—1α (IL-1α) and epidermal growth factor (EGF) [75]. Under controlled conditions, copper significantly contributes to healing by enhancing the expression of extracellular matrix molecules, such as fibrinogen, collagen formation, and integrins, which are the key mediators of cell attachment to the extracellular matrix [76]. However, CuNPs can accumulate in the body or release excessive ions, potentially causing long-term toxicity or generating free radicals, leading to lipid peroxidation and cell death [77]. Controlled Cu^2+^ ion release can reduce toxicity levels while enhancing cell migration, angiogenic capabilities, and active wound healing [78]. In diabetic wounds, this multifaceted role of copper in wound healing and tissue repair highlights its potential therapeutic applications.

### 4.2. Zinc

Zinc is an essential trace element for human health, contributing to physical development and the maintenance of musculoskeletal integrity [79]. As a cofactor for nearly 3000 proteins and enzymes, zinc is crucial for neural function, growth and development, bone mineralization, immune response and wound healing [80]. Zinc also affects hemostasis by influencing coagulation factors, platelet aggregation, and interaction with endothelial cells, enhancing clot stability by accelerating clot formation [81]. The antibacterial properties of zinc oxide nanoparticles may depend on their size, which can affect their ability to disrupt bacterial cell membrane and induce oxidative stress [82]. Furthermore, its anti-inflammatory and angiogenic properties have demonstrated significant therapeutic benefits in wound healing [83]. As a result, Zn^2+^ can promote self-cleaning and keratinocyte migration during wound healing, contributing to skin regeneration [84].

### 4.3. Magnesium

The importance of magnesium in biomedical applications is linked to its essential role in cellular process and human metabolism [85]. Mg^2+^ is involved in various physiological activities, either alone or alongside biomolecules. It serves as an enzyme activator, aiding processes such as glycolysis, fatty acid oxidation, bone formation, and neuromuscular excitability [86]. Magnesium ions can influence inflammatory and immune responses, in addition to controlling various cellular functions, including adhesion and proliferation [87]. Moreover, Mg^2+^ supports angiogenesis, a vital process in wound healing that restores oxygen and nutrients to tissues undergoing regeneration [88]. Mg^2+^ plays a pivotal role in a range of cellular responses, including cell proliferation, cell cycle, and protein synthesis, through both direct and indirect mechanisms [89].

### 4.4. Silver

Silver ions and silver nanoparticles (AgNPs) have attracted considerable attention across biomedical, engineering, and environmental fields owing to their unique physicochemical properties and broad-spectrum antimicrobial activity. They are commonly used in antibacterial cleaning agents, sterilization, and disinfection detergents for medical devices, as well as drug delivery systems [90]. The exceptional antimicrobial properties of AgNPs have been extensively validated, with bactericidal activity reported against over 500 pathogenic microorganisms. This broad-spectrum efficacy, encompassing Gram-positive, Gram-negative, and drug-resistant pathogens, has positioned AgNPs as one of the most widely investigated antimicrobial nanomaterials for biomedical applications [91]. AgNPs facilitate the transition from the inflammatory phase to the proliferative phase of diabetic wounds, thus enhancing their healing process [92]. The antimicrobial and antitumoral efficacy of AgNPs is largely associated with their capacity to generate reactive oxygen species (ROS), resulting in oxidative stress-mediated disruption of cellular respiration and damage to essential cellular components. In cancer cells, AgNP-induced ROS accumulation has been reported to trigger cytotoxic responses, including DNA damage, cell-cycle arrest, inhibition of proliferation, and activation of apoptotic pathways, ultimately leading to cell death [93]. Despite this pro-oxidant activity towards pathogens and malignant cells, AgNPs have also demonstrated notable antioxidant capacity in radical-scavenging assays when compared to the standard use. Furthermore, plant-derived AgNP-containing wound dressings have demonstrated enhanced regenerative capacity, characterized by increased collagen deposition, accelerated healing, and improved tissue remodeling, thereby supporting more effective wound repair than conventional approaches [94].

### 4.5. Manganese

Manganese (Mn) is a vital element involved in numerous biological processes and serves as a cofactor for enzymes such as arginase, prolidase, and RNA polymerase [95]. Recently, Mn-based nanozymes have attracted considerable attention as catalytic antioxidants capable of mimicking the activities of superoxide dismutase (SOD), catalase (CAT), and glutathione peroxidase (GPx). Through efficient scavenging of reactive oxygen species (ROS) and restoration of intracellular redox balance, Mn^2+^-containing systems mitigate oxidative stress and suppress excessive inflammatory responses [96]. Manganese ions have been reported to stimulate keratinocyte and fibroblast growth by promoting integrin expression during the proliferation phase on in vivo monolayer studies [97]. Mn^2+^ plays a multifaceted role in wound repair by facilitating proline synthesis, a key prerequisite for collagen deposition and extracellular matrix formation. In addition, Mn^2+^ promotes re-epithelialization by activating integrin-dependent signaling pathways, thereby enhancing cell adhesion, migration, and tissue regeneration [98]. Despite its multifunctional biological activity and promising regenerative potential, manganese remains one of the least investigated therapeutic ions in wound-healing research, and further studies are required to fully elucidate its mechanisms of action and therapeutic benefits [99].

The therapeutic ions discussed in this review, namely Cu^2+^, Zn^2+^, Mg^2+^, Ag^+^, and Mn^2+^, were chosen because of their recognized importance in biological processes related to tissue repair and wound healing. While Ag^+^ is extensively utilized in clinical settings for infection management, the other ions naturally occur in the human body and are involved in crucial physiological functions, such as angiogenesis, collagen production, enzyme regulation, antioxidant protection, and cell proliferation [100]. Moreover, these ions are cost-effective, easily accessible, and can be seamlessly integrated into biomaterial platforms, making them promising options for creating clinically applicable and economical wound dressings [101]. Although metals such as gold, selenium, and cobalt have shown potential therapeutic benefits, their high costs, possible toxicity, limited natural occurrence, and reduced translational potential may hinder their widespread use, especially in resource-constrained environments [102]. Consequently, this review focuses on therapeutic ions that combine biological relevance with economic feasibility, thereby supporting the development of cost-effective and accessible strategies for diabetic wound management.

### 4.6. Synergistic Integration of Multiple Therapeutic Ions Within a Single System

Although copper, zinc, magnesium, silver, and manganese have traditionally been investigated as individual therapeutic agents, increasing attention has been directed toward their integration within multifunctional biomaterial platforms to harness potential synergistic effects. In particular, chitosan-based systems are being engineered to accommodate multiple therapeutic ions within a single matrix, enabling the simultaneous delivery of complementary biological functions. For instance, copper is frequently combined with zinc or silver to integrate the pro-angiogenic activity of copper with the potent antibacterial properties of zinc or silver [103]. Similarly, magnesium and manganese are typically used alongside copper or zinc to promote cell growth and provide antioxidant protection without increasing the antimicrobial burden [104]. The incorporation of multiple therapeutic ions into a single biomaterial platform presents significant opportunities for enhancing wound-healing outcomes but requires careful optimization to ensure therapeutic efficacy and biological safety. Excessive ion concentrations may compromise beneficial antimicrobial, angiogenic, and regenerative effects by inducing cytotoxicity, oxidative stress, or impaired tissue repair. Therefore, precise control over ion composition, concentration, and release behavior is essential for maximizing therapeutic performance [105]. Despite growing interest in multi-ion-functionalized biomaterials, the mechanisms governing ion-ion interactions, synergistic and antagonistic effects, and their collective biological impact remain poorly understood. Further research is needed to establish quantitative design principles that will guide the development of safe, effective, and clinically translatable multi-ion wound-healing systems [106].

### 4.7. Safety Considerations and Limitations Associated with Therapeutic Ions

As summarized in Table 3, the acceptable daily intake (ADI) values established by the European Food Safety Authority (EFSA) are reported alongside the locally safe topical concentrations for each ion. It should be noted that ADI values conventionally refer to systemic oral exposure, whereas the topical concentrations reported here reflect local, wound-site application; the two routes differ in absorption kinetics and are not directly interchangeable but are presented together to provide a broader safety context for both local and potential systemic exposure. Chronic, high-dose systemic exposure to silver has been associated with argyria, a bluish-gray skin discoloration resulting from silver deposition in tissues. In contrast, the topical concentrations considered herein are localized and administered for limited periods, substantially reducing the risk of such adverse effects [107]. Likewise, although Cu^2+^ promotes angiogenesis and cell proliferation within an appropriate therapeutic window reported in Table 3, excessive or prolonged copper exposure has been associated with oxidative stress and hepatotoxicity, highlighting the importance of controlled and localized dosing strategies in copper-based wound therapies [74]. In vitro cytotoxicity assessments on human keratinocytes and fibroblasts, the primary cell types involved in wound re-epithelialization and dermal repair, are essential for defining ion-specific safety thresholds and should be prioritized in future investigations [108].

Despite their considerable therapeutic potential, ion-releasing biomaterials remain limited by the need for precise control over ion delivery. Excessive local ion concentrations or prolonged exposure may compromise biocompatibility and healing outcomes, narrowing the therapeutic window of metal-based systems [105]. Beyond local biocompatibility, the long-term systemic fate of metal-ion-based therapies remains an important consideration. Although localized administration is expected to minimize systemic exposure, released metal ions or metal-containing nanomaterials may migrate from the treatment site, enter systemic circulation, and accumulate in secondary organs. Particular attention should be paid to the liver, spleen, kidneys, and brain because of their roles in metal uptake, clearance, and homeostasis [109]. Therefore, the absence of local toxicity does not necessarily guarantee long-term systemic safety. Comprehensive safety assessments should include not only histological evaluation of the treated tissue but also metal biodistribution, metabolic clearance, biochemical markers, urinary excretion, and long-term retention profiles of residual metal species [110].

**Table 3:** A comparative table of therapeutic ion properties: concentration, main function, biocompatibility, antibacterial mechanism and acceptable daily intake (ADI) levels. * Safe dose and concentration for topical application. ** Approximate value of the ADI (established by EFSA for adults) in body weight (bw) per day.

Nanoparticle	Safe Dose *	Ion Function in Biological System	Biocompatibility	Antibacterial Mechanism	ADI Value **	Ref
Copper NPs (Cu^2+^)	40 nm– 80 nm ≤2 mg/mL	Acts as a stimulant for the proliferation of human endothelial cells. Stimulates cells to release growth factors and cytokines. Antibacterial effects. Activates endothelial growth factor and increases angiogenesis stimulation in combination with FGF-2.	No deleterious effects on living cells, CuNPs concentration of 35 nmol/mg shows more biocompatibility	Cu^2+^ generates hydroxyl radicals to attack the bacterial membrane.	0.07 mg/kg bw/day	[111,112]
Zinc Oxide NPs (Zn^2+^)	15 nm– 55 nm 1.0 mg/mL–10 mg/mL	Zinc ion regulates the activity of growth hormone. Promotes neural development Improves osteoblast differentiation to promote bone development. Exhibits anti-inflammatory properties. Prevents bacterial development and facilitates wound healing at the surgical site.	FDA recognized non-harmful material; widely used in biomedical imaging, biosensing, gene delivery, and drug delivery	ROS production, lipopolysaccharide membrane rapture, DNA replication inhibition, and lowering the bacteria’s enzymatic metabolism.	25 mg/day	[113,114]
Magnesium hydroxide NPs (Mg^2+^)	250 and 500 μg/mL	Acts as a coagulant, cofactor for a variety of enzymes (catalytic action), usually exists as a chelate in cells.	Considered safe in vivo and in vitro at lower concentrations; safe at <250 μg/mL suitable for drug encapsulation, water purification and clinical uses. Higher concentrations may lead to cancer.	Membrane destabilization; disruption of Mg^2+^-dependent enzymatic/metabolic processes; inhibition of bacterial adhesion and biofilm formation.	350 mg/day (men) 300 mg/day (women)	[115,116]
Silver NPs (Ag^+^)	≥10 nm 1.0 mg/mL–3.0 mg/mL	Exerts antibacterial activity through interactions with microbial DNA, inhibiting replication, and with sulfhydryl-containing bacterial enzymes, disrupting cellular respiration and membrane-associated transport processes. Widely employed in the development of bioactive and antimicrobial bone implants owing to their broad-spectrum antimicrobial activity and regenerative potential.	Used in wound dressings, safe at lower concentrations	Ag^+^ interacts with sulfur-containing proteins in the bacterial cell membrane.	0.9 μg/kg bw/day	[117,118]
Manganese (Mn^2+^)	2 ppm–256 ppm	A cofactor for a wide range of enzymes (oxidoreductases, transferases, hydrolases, lyases, isomerases, ligases, lectins, integrins, and glutamine synthetase). Superoxide-free radical detoxification is impossible without it. Used as an MRI contrast agent. Antibacterial and bio-tolerant properties.	Low-dose antibacterial efficacy, antioxidant data, and compatibility with antibiotics.	Decreasing the level of ROS, suppressing the inflammation and neutrophil infiltration, and promoting the polarization of macrophages into M2-type.	3 mg/day	[119,120]

Consequently, the rational design of next-generation ion-functionalized biomaterials requires achieving an optimal balance between antimicrobial activity, tissue regeneration, immune modulation, and long-term safety. Future research should focus on three key areas. First, the development of precisely regulated ion-delivery systems capable of maximizing therapeutic efficacy while minimizing toxicity [121]. Advances in nanotechnology, smart biomaterials, and stimuli-responsive platforms offer promising opportunities to achieve controlled ion release tailored to the dynamic requirements of different stages of wound healing. Second, the interactions between therapeutic ions and biomaterial matrices, particularly in multi-ion systems, remain insufficiently understood. Emerging evidence suggests that combining multiple ions may generate synergistic effects that enhance healing outcomes, therefore, future studies should optimize ion combinations and elucidate the mechanisms governing their release, bioavailability, and biological activity in different wound environments [122]. Finally, comprehensive long-term safety evaluations are essential for clinical translation. Beyond the scope of local histocompatibility, assessments should include systemic biodistribution, organ accumulation, metabolic clearance, immune responses, degradation products, and potential disruptions of metal homeostasis [123]. These considerations are especially important for slowly degradable or persistent nanosystems, such as iron oxide-based scaffolds and catalytic nanozymes, which may remain in neural tissue or peripheral organs for extended periods [124]. Accordingly, future investigations should integrate histological analyses, blood biochemistry, organ pathology, and long-term monitoring of metal retention and clearance to establish the safety and therapeutic durability of these advanced wound-healing platforms.

## 5. Potential of Mesoporous Silica Nanoparticles (MSNs) for Enhanced Wound Healing in Diabetes

### 5.1. Properties of MSNs

Mesoporous silica materials have emerged as notably promising platforms for wound-healing applications [125,126] due to their distinctive structural features, biocompatibility, and remarkable drug-loading capabilities [38]. These materials are distinguished by their highly porous structure, featuring narrow pore size distributions with diameters ranging from 2 to 50 nm, large pore volumes, and exemplary specific surface areas, typically between 300 and 1000 m^2^g^−1^. Representative MSNs, including SBA-15 [127] and MCM-41 [128] exhibit ordered porosity with a two-dimensional (2D) hexagonal pore architecture composed of uniform cylindrical mesochannels, enabling efficient mass transport and precise control over drug loading and release kinetics. The outstanding chemical, hydrothermal, and mechanical stability of MSNs, along with the superior tunability of their physicochemical properties, including morphology, particle size, pore architecture and surface functionality, allow the development of customized therapeutic delivery strategies for advanced biomedical applications [129,130]. MSNs, particularly SBA-15 [127,131], are highly versatile materials whose surfaces can be readily functionalized with a broad spectrum of organic moieties, including amine, thiol, carboxyl, and phosphate groups. In addition, surface engineering enables precise modulation of their characteristics, leading to increased drug-loading efficiency, improved control over drug-release profiles, enhanced solubility and bioavailability of poorly water-soluble therapeutics. It also provides effective protection for sensitive bioactive cargos, including antibiotics, growth factors, peptides, nucleic acids, enzymes, and imaging agents, thereby minimizing premature degradation and preserving their biological activity. Moreover, their well-defined mesoporous architecture facilitates localized and sustained delivery of these therapeutic agents, enhancing treatment efficacy while reducing systemic exposure and associated side effects [132,133,134].

The preservation or generation of silanol-rich surfaces represents a critical aspect in the development of MSNs, as surface silanol groups are directly responsible for many of their advantageous physicochemical and biological properties. These functionalities govern surface reactivity, hydrophilicity, biomolecular interactions, drug-loading efficiency, and subsequent functionalization processes. Consequently, maintaining a high density of accessible silanol groups is essential for tailoring MSN performance and can be effectively obtained through solvent extraction-based surfactant removal methods, which preserve the surface chemistry of the silica framework while minimizing silanol condensation during processing [135,136,137].

Overall, the high degree of structural and surface tunability of MSNs constitutes a major advantage, enabling the rational engineering of nanoplatforms with enhanced biocompatibility, bioactivity, and cellular interactions while reducing potential cytotoxicity. Such modifications can significantly improve therapeutic efficacy and promote regenerative processes, underscoring the potential of MSNs for advanced biomedical and tissue-engineering applications [126,129,138].

### 5.2. Hybrid Platforms Incorporating MSNs

The incorporation of MSNs into formulations containing polymers, ceramics, metals or other nanomaterials enables the integration of complementary functionalities into a single platform, broadening their potential for biomedical applications. Palanikumar, et al. [139] developed a polymer-gatekeeper MSNs where doxorubicin loaded-MSNs were non-covalently capped with a disulfide-cross linkable PEG-PDS copolymer to fabricate a targeted drug delivery system. Wu, et al. [140] synthesized a nanocomposite by immobilizing ultrasmall ceria nanocrystals onto MSN surfaces, combining strong tissue adhesion through nano bridging with potent autocatalytic ROS-scavenging activity. In a rat model, MSN-ceria treatment not only accelerated wound closure but promoted regenerative healing characterized by skin appendage morphogenesis, native collagen alignment and limited scar formation. This MSN-based nanocomposite showed promise in modulating the oxidative stress microenvironment particularly relevant to impaired diabetic wound healing. Xue, et al. [141] incorporated artemisia argyi extract-loaded MSNs into a GelMA/HAMA photocrosslinkable hydrogel, where the mesoporous carriers dramatically suppressed burst release. The resulting composite hydrogel exhibited antibacterial activity, promoted M1-to-M2 macrophage polarization, and significantly accelerated wound closure with enhanced collagen deposition. Studies in angiogenesis using rat full-thickness wound models have demonstrated the effectiveness of MSNs as sustained-release delivery systems, enhancing therapeutic efficacy in chronic wound healing. Zhang, et al. [142] specifically designed a dual-drug loaded composite hydrogel dressing comprising oxidized hyaluronic acid, branched polyethyleneimine and amino-modified mesoporous silica, enabling sustained tetracycline delivery and in situ metformin incorporation via Schiff-base crosslinking for diabetic foot ulcer treatment. Their findings highlighted the capacity of MSN-integrated hydrogel dressings to provide intelligent, context-responsive multi-drug delivery to simultaneously address the complex pathological features of diabetic wounds. The key outcomes discussed above are summarized in Table 4.

**Table 4:** Main outcomes of these representative mesoporous silica-based drug delivery systems for wound healing.

Mesoporous System	Therapeutic Agent	Main Outcomes	Ref
Polymer-gatekeeper MSN, non-covalently capped with a disulfide-crosslinkable PEG-PDS copolymer	Doxorubicin	High drug loading was achieved because the unmodified pore volume was preserved	[139]
Ultrasmall ceria nanocrystals immobilized on MSN surfaces	Autocatalytic ROS-scavenging ceria (no exogenous drug)	Accelerated wound closure, regenerative healing with skin appendage formation, native collagen alignment and limited scarring	[140]
MSNs incorporated into GelMA/HAMA photocrosslinkable hydrogel	Artemisia argyi extract	Antibacterial activity, M1-to-M2 macrophage polarization, accelerated closure with enhanced collagen deposition and angiogenesis	[141]
Amino-modified MSNs within an oxidized hyaluronic acid/branched polyethleneimine hydrogel	Tetracycline and metformin (dual-drug, Schiff base crosslinked)	Successful multi-drug encapsulation enabling responsive release	[142]

Despite significant advances in diabetic wound research, the heterogeneity of biomaterials, therapeutic cargoes, and experimental models used across studies limits rigorous quantitative comparison and standardization of outcomes.

## 6. Superparamagnetic Iron Oxide Nanoparticles (SPIONs) for Diabetic Wound Healing

### 6.1. Properties of SPIONs

Superparamagnetic iron oxide nanoparticles (SPIONs) have gained increasing research interest for biomedical applications. Owing to their capabilities and magnetic responsiveness, SPIONs act as multifunctional theranostic platforms, enabling targeted and controlled drug delivery, high-resolution MRI contrast enhancement, cellular tracking, and magnetic hyperthermia-mediated therapy [143,144,145,146]. SPIONs belong to the class of ferrimagnetic iron oxide nanomaterials, primarily represented by magnetite (Fe_3_O_4_) and maghemite (γ-Fe_2_O_3_), although mixed ferrites (MFe_2_O_4_, where M = Co, Mn, Zn, etc.) have also been extensively investigated due to their tunable magnetic properties. Their distinctive superparamagnetic behavior, which arises when particle dimensions are reduced to the nanoscale (typically 10–50 nm), enables precise remote manipulation through the application of an external alternating magnetic field. Under these conditions, the magnetic moments within the nanoparticles rapidly align with the applied field and lose their magnetization upon its removal, thereby preventing magnetic remanence and reducing particle aggregation [147]. Their large specific surface area provides abundant sites for surface functionalization, covalent attachment or loading of biological targeting moieties [144]. This versatility facilitates the development of targeted nanoplatforms with improved biodistribution, enhanced target-site recognition, and increased therapeutic efficacy while minimizing off-target effects [146,148]. Moreover, SPIONs provide efficient magnetic resonance imaging (MRI) contrast, allowing precise visualization and discrimination of pathological and healthy tissues. In addition to their unique magnetic properties, SPIONs offer several advantages, exhibiting favorable biodegradability and biocompatibility, enabling their safe interaction with biological systems and facilitating a wide range of therapeutic and diagnostic functions [149]. In the field of oncology, SPIONs have revealed significant outcomes as therapeutic agents, particularly when activated by externally applied alternating magnetic fields. This approach offers several notable advantages, including minimal invasiveness, reduced dependence on the physicochemical characteristics of the surrounding tissue, and the absence of penetration-depth limitations typically associated with conventional therapeutic modalities. Consequently, SPION-based systems offer an effective treatment for deep-seated malignancies and have attracted considerable interest in brain tumor therapy, where their magnetic guidance and targeting capabilities may facilitate drug delivery across the blood–brain barrier. Furthermore, their ability to generate localized heat under alternating magnetic fields provides a suitable platform for magnetic hyperthermia, enhancing antitumor efficacy while minimizing damage to healthy tissues [145,150,151]. The significant translational potential of SPIONs has led to the clinical approval of several SPION-based formulations by the FDA [152]. More recently, these nanomaterials have attracted growing attention in wound-healing research owing to their capacity to modulate the wound microenvironment, enhance tissue repair processes, and stimulate regeneration in chronic wounds [143,153].

Despite all their advantages, uncoated SPIONs exhibit high surface energy and chemical reactivity due to their elevated surface-to-volume ratio, making them prone to oxidation under physiological or ambient conditions and to the formation of aggregates. Such phenomena can significantly compromise their colloidal stability, magnetic performance, and biological effectiveness. Consequently, surface modification has become a critical strategy for enhancing SPION functionality, as it improves physicochemical stability, prevents aggregation, increases biocompatibility, and enables the introduction of additional functionalities tailored to specific biomedical applications [154]. To this end, a wide variety of organic and inorganic coating approaches, as well as the development of hybrid and composite nanostructures, have been proposed to optimize the performance of SPION-based theranostic platforms [39].

### 6.2. Potential Role of SPIONs in Diabetic Wound Healing

Numerous studies establish SPIONs as multifaceted platforms capable of integrating infection control, cellular modulation, targeted therapeutic delivery, and real-time monitoring across a broad range of wound-healing applications. Notably, many of these functionalities directly address the pathological hallmarks of diabetic wounds, which differ substantially from acute wounds and are characterized by chronic inflammation, excessive oxidative stress, persistent bacterial infection, impaired angiogenesis, tissue hypoxia, and delayed cellular responses [155,156]. In this context, SPIONs can be remotely manipulated using external magnetic fields, enabling non-invasive stimulation, targeted delivery, and imaging of therapeutics within the wound microenvironment. Unlike optical-based techniques, magnetic fields exhibit excellent tissue penetration and are not significantly affected by the poor vascularization and compromised blood perfusion that characterize diabetic wounds [144,157]. Furthermore, the large surface area and versatile surface chemistry of SPIONs allow functionalization with bioactive molecules, targeting ligands, growth factors, antimicrobial agents, and regenerative therapeutics, facilitating the development of multifunctional platforms tailored to the complex needs of diabetic wound management [158]. Their iron-based composition may provide additional biological benefits, as iron oxide nanoparticles have been reported to influence cellular signaling pathways involved in angiogenesis, macrophage polarization, and tissue remodeling, effectively addressing the impaired angiogenesis and dysregulated inflammation that characterize the diabetic wound microenvironment, in a way that is difficult to achieve with static, non-responsive materials [153]. Iron oxide in SPIONs is processed through the body’s natural iron pathways, which is beneficial for patients who already struggle with impaired wound healing and have a reduced capacity to handle additional foreign substances [151]. Consequently, the unique combination of magnetic properties and regenerative potential renders SPIONs particularly attractive candidates for the treatment of chronic diabetic wounds, where conventional therapeutic approaches often show limited effectiveness.

### 6.3. Hybrid Platforms Containing SPIONs for Wound Healing and Theranostic Applications

Guo, et al. [159] proposed SPION-based scaffolds with dual functional potential in wound-associated tissue regeneration. They demonstrated that Feraheme-like SPIONs assembled layer-by-layer onto 3D-printed PLGA scaffolds conferred both antibacterial and immunomodulatory properties. The authors concluded that the regenerative effects of SPIONs were primarily attributed to their unique surface characteristics rather than solely to the release of iron ions resulting from nanoparticle degradation. These findings highlight the crucial role of surface-mediated biointeractions in modulating cellular responses and promoting tissue regeneration, thereby providing a promising strategy for the design of infection-responsive scaffolds for wound-healing applications. Liu, et al. [160] revealed significant potential of SPIONs when applied to nerve wound healing through a mechanism they described as SPION-mediated magnetic actuation. Given the central role of Schwann cells in peripheral nerve regeneration, and their progressive loss of repair-associated phenotypes following injury, strategies aimed at maintaining their regenerative capacity are of considerable clinical interest. To address this challenge, they developed fluorescent-magnetic bifunctional SPIONs and demonstrated that, in the presence of an external magnetic field gradient, these nanoparticles could exert piconewton (pN)-scale mechanical forces on Schwann cells. The applied mechanical stimuli were subsequently converted into intracellular biochemical signals through mechanotransduction pathways, leading to the upregulation of key regeneration-associated transcription factors. Their findings indicated that SPIONs can function as effective mechanotransduction actuators, capable of preserving the repair-supportive phenotype of Schwann cells. This approach therefore represents a promising therapeutic strategy for enhancing peripheral nerve regeneration and improving outcomes in nerve injury repair. Recently, Hashemi, et al. [161] proposed that SPIONs serve a dual role in wound healing, functioning both as cell-labeling agents for the non-invasive tracking of transplanted stem cells and as facilitators of regenerative cell therapy delivery. Simultaneously, their integration within stem cell-based constructs enhances the precision and effectiveness of cell delivery to the target tissue. Consequently, SPIONs represent an invaluable theranostic platform in wound healing, combining therapeutic functionality with non-invasive imaging capabilities. This dual functionality offers a powerful approach for stem cell-based regenerative therapies, thereby opening new avenues for advanced wound management and regenerative medicine.

Vargas-Osorio, et al. have made significant contributions to the development of multifunctional magnetic nanocomposites by integrating SPIONs (Fe_3_O_4_) within SBA-15 mesoporous silica frameworks [162,163,164,165] or by engineering functionalized multicore-shell (multi-Fe_3_O_4_@C-COOH) nanostructures [166] for advanced theranostic applications. This synergistic hybridization strategy has demonstrated considerable promise for applications involving brain, and soft- and hard-tissue regeneration. Among their most notable properties, these magnetic mesoporous nanocomposites exhibit highly ordered porosity and large specific surface areas. The magnetic SBA-15 structures generally preserve the characteristic two-dimensional hexagonal mesostructure with uniform cylindrical pores, while magnetite nanoparticles are covalently anchored to the silica surface, enabling a strong magnetic response even at relatively low iron oxide content. Furthermore, these materials retain the superparamagnetic behavior of SPIONs, characterized by the absence of magnetic remanence and coercivity, thereby minimizing undesirable particle aggregation. Their thermal-insulating capability during magnetic stimulation limited excessive temperature increases and helped prevent thermal damage to surrounding healthy tissues. This characteristic is particularly relevant for applications involving sensitive organs, such as the brain, where strict thermal control is essential. In addition, the synthesis approaches allowed precise control over particle morphology, enabling the fabrication of nanostructures with tailored sizes and shapes according to the requirements of specific biomedical applications. The abundance of surface silanol groups further contributes to enhanced colloidal stability, biocompatibility, and bioactivity in physiological environments, while providing versatile anchoring sites for subsequent functionalization. From a diagnostic perspective, these nanocomposites have demonstrated excellent performance as MRI contrast agents, exhibiting high transverse relaxivity (r_2_) values that are comparable to or even surpass those commercially available, thereby improving imaging sensitivity and diagnostic accuracy. Moreover, the simultaneous incorporation of magnetic nanoparticles and fluorescent probes, such as rhodamine derivatives, enables multimodal MRI/optical imaging, allowing more comprehensive visualization and tracking of biological processes. Collectively, these physicochemical, magnetic, and imaging characteristics highlight the considerable potential of magnetic SBA-15/SPION nanocomposites as multifunctional theranostic platforms. Their ability to combine targeted therapeutic delivery, non-invasive imaging, high biocompatibility, and magnetic responsiveness makes them particularly attractive candidates for diabetic wound-healing applications, where the need for real-time therapeutic monitoring pose significant clinical challenges.

### 6.4. Diagnostic and Monitoring Capabilities of SPIONs Through Magnetic Resonance Imaging (MRI)

MRI is well known for its excellent soft tissue contrast and ability to penetrate tissues without restriction [167]. However, its relatively low spatial resolution and lack of real-time imaging capabilities make it less ideal for directly assessing superficial wound beds compared with optical-based methods. In contrast, fluorescence and near-infrared (NIR) imaging offer significantly higher spatial resolution at shallow tissue depths, allowing non-invasive, real-time visualization of nanoparticle distribution and related biological responses [168]. Photoacoustic imaging adds another layer by enabling the functional evaluation of wound vascularization and tissue oxygenation through the combined detection of optical absorption and ultrasonic signals [169]. Owing to these complementary advantages, SPION-containing mesoporous silica nanoparticle (MSN) hybrids are appealing because they are not limited to a single imaging technique. The high surface area and adjustable surface chemistry facilitate the dense and controlled attachment of fluorophores, targeting antibodies, and other responsive probes [170]. Meanwhile, the superparamagnetic iron oxide nanoparticle (SPION) component, whether integrated within the mesoporous structure or as a magnetic core, provides MRI contrast enhancement and magnetic responsiveness. Structures such as SBA-15/Fe_3_O_4_ composites have been demonstrated to mitigate the aggregation commonly observed in bare magnetite nanoparticles while preserving a highly functionalizable surface [171]. Furthermore, surface amination using (3-aminopropyl) triethoxysilane (APTES) provides reactive amino groups that enable the covalent conjugation of fluorescent probes, such as rhodamine derivatives, as well as targeting biomolecules, thereby expanding the diagnostic and therapeutic capabilities of Fe_3_O_4_-containing silica platforms [171]. This surface chemistry underpins the development of antibody-functionalized magnetic mesoporous silica hybrids for targeted and image-guided drug delivery. Beyond enabling dual-modal fluorescence/MRI imaging, SPIONs provide additional functionalities relevant to wound management, including magnetically assisted retention at the wound site, externally triggered or magnetically modulated therapeutic release, and non-invasive monitoring of nanoparticle biodistribution and clearance beyond the limitations of optical imaging [172]. Consequently, MSN-SPION nanocomposites outperform conventional contrast agents by acting as multifunctional theranostic platforms that integrate complementary imaging capabilities with the sustained release of therapeutic agents, thereby offering significant potential for future image-guided wound management.

## 7. New Generation of Multifunctional Theranostic Chitosan-Based Dressings for Diabetic Wound Healing

To address the complex requirements of diabetic wounds, a single-component dressing is unlikely to provide an effective solution. While Section 2, Section 3, Section 4, Section 5 and Section 6 demonstrated the potential of chitosan, therapeutic ions, MSNs and SPIONs as individual functional components, the real translational value emerges only when all four components are brought together within a single, rationally engineered system. No single component can simultaneously address all the factors that define the diabetic wound microenvironment. The present review emphasizes the rational integration of these four components into a unified multifunctional platform capable of addressing the complex and multifactorial nature of diabetic wounds. To the best of our knowledge, no studies have yet described the combination of all these components within a unified platform for chronic or diabetic wound healing. Moreover, their application remains relatively unexplored across the broader biomedical field despite the complementary functionalities and versatility offered by each constituent. This gap in the literature highlights both the originality of the proposed approach and its significant potential for the development of advanced multifunctional systems with therapeutic and diagnostic capabilities, as illustrated in Figure 3.

**Figure 3:**
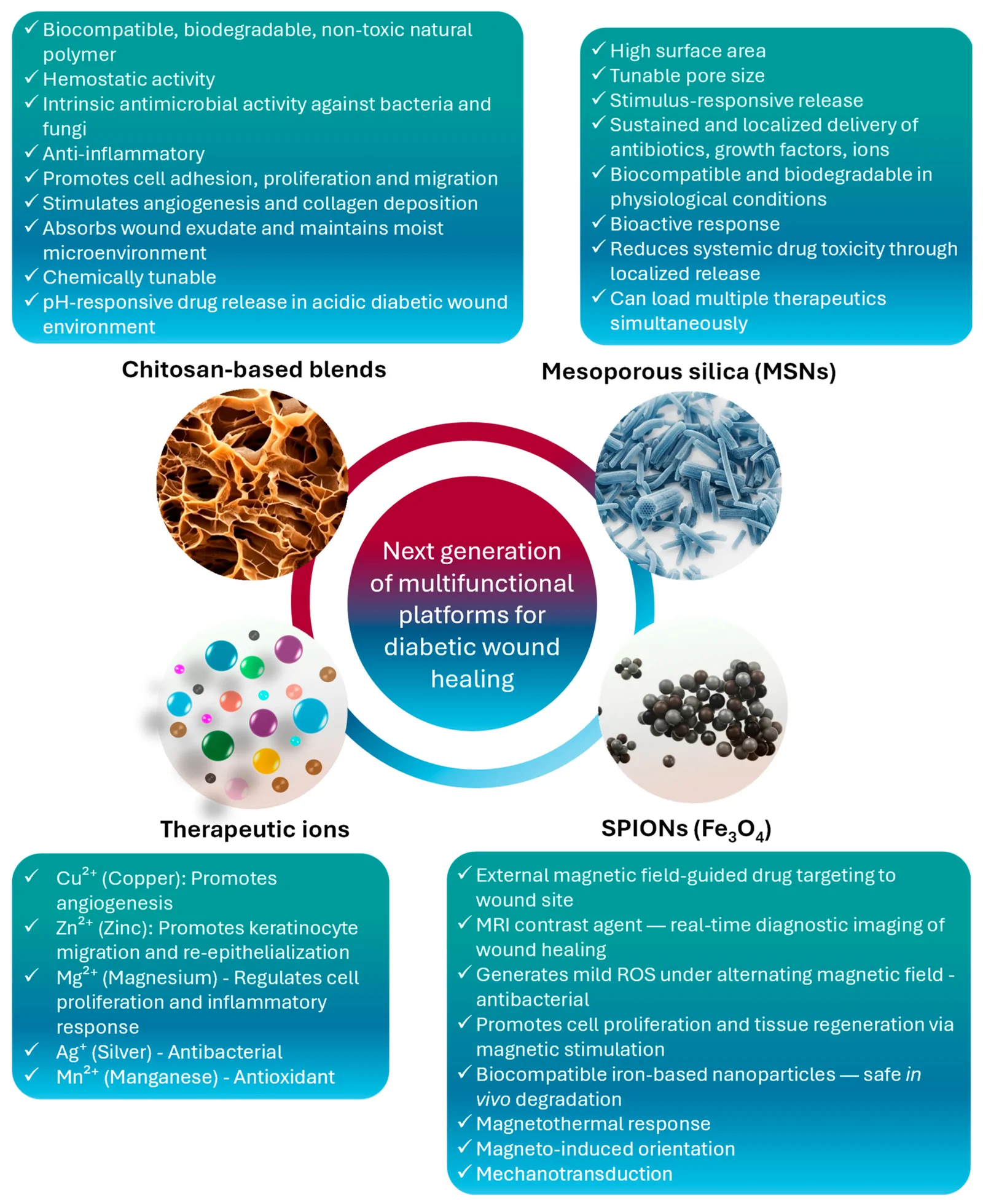
Overview of the individual contributions of each component and their synergistic integration within the proposed multifunctional theranostic platform for diabetic wound management. Parts of this figure were created with the assistance of artificial intelligence (AI) tools and were subsequently reviewed and edited by the authors.

Importantly, the modular nature of this platform enables extensive tuning of its structural and physicochemical properties, facilitating the fabrication of a wide range of constructs, such as sponges, hydrogels, films, beads, fibers and microgels. This design flexibility allows the development of application-specific systems tailored to clinical needs, including efficient exudate management, adaptation to irregular wound topographies, sustained therapeutic delivery, and injectable formulations for minimally invasive treatment.

This section of the review focuses on the outcomes and synergistic interactions identified in a series of studies conducted by Vargas-Osorio, et al. [57,173,174,175,176] which provide compelling evidence supporting the feasibility of the integrated strategy proposed herein. These findings not only validate the underlying design principles of the proposed system but also establish a strong foundation for extending this approach to diabetic wound-healing applications, where similarly complex therapeutic challenges must be addressed simultaneously. Figure 4 summarizes the properties achieved through the integration of at least three of the four components discussed in this review, as demonstrated in multifunctional hybrid sponges developed for bone tissue engineering.

**Figure 4:**
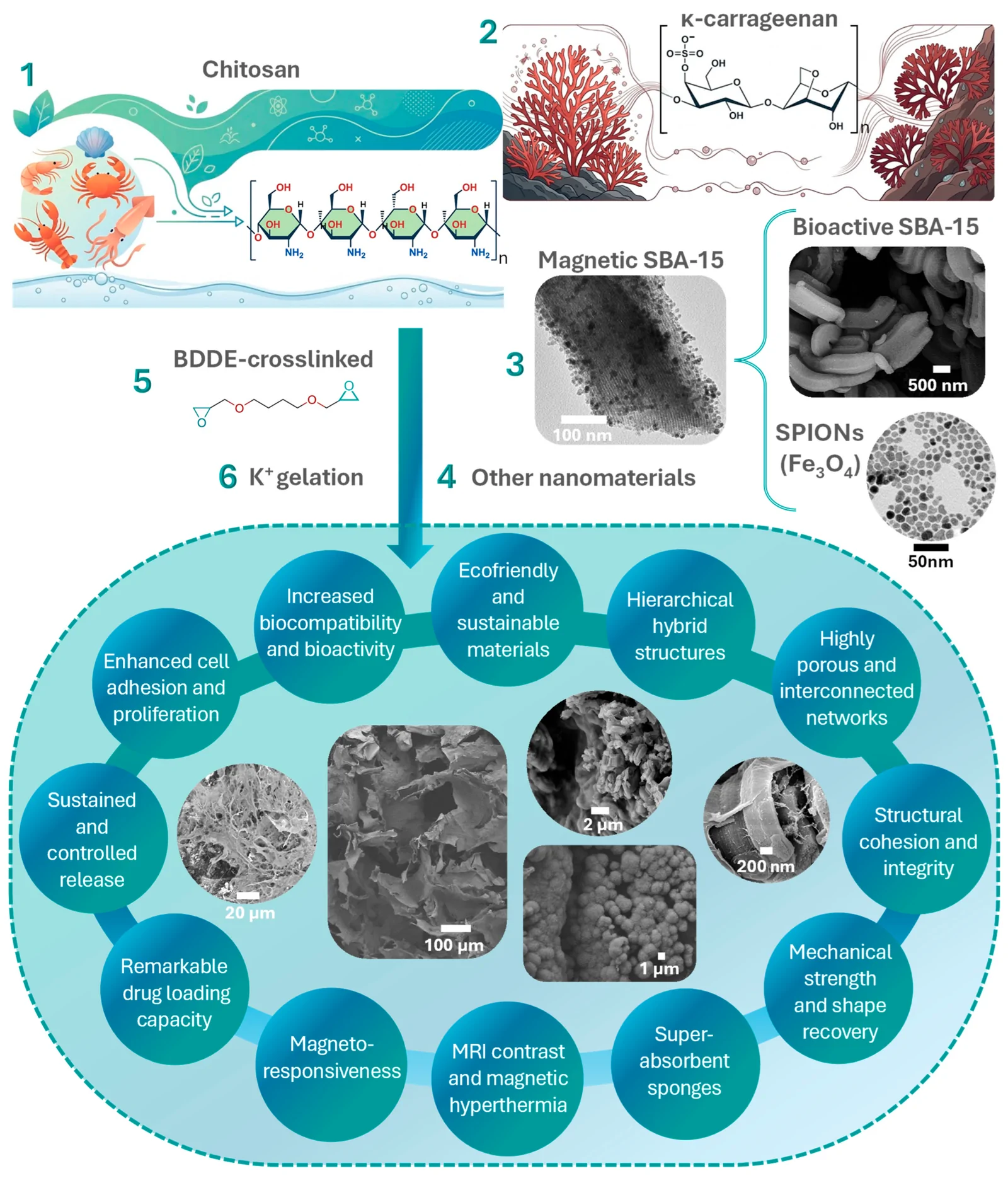
Summary of the functional properties achieved through the integration of multiple components in multifunctional hybrid sponges. Parts of this figure were created with the assistance of artificial intelligence (AI) tools and were subsequently reviewed and edited by the authors.

### 7.1. Sustainable and Environmentally Safe Fabrication of the Theranostic Platform

The potential of marine-derived polysaccharides—chitosan (CS) and kappa-carrageenan (κ-C)—as natural, renewable, and environmentally sustainable biomaterial sources were highlighted through the successful fabrication of various multifunctional architectures, including nanofibers [57,173] and three-dimensional (3D) hybrid sponges [174,175,176]. SBA-15 nanoparticles or engineered SBA-15@Fe_3_O_4_ composites, and in some cases, a combination of these systems with other inorganic materials such as nanohydroxyapatite (n-HAp) were additionally integrated to the sponges. For their fabrication, environmentally friendly solvents, such as water, formic acid, and acetic acid were employed as safer alternatives to the toxic organic solvents commonly used in conventional processing methods. Such approaches are consistent with the principles of green chemistry. Furthermore, the synthetic and processing methodologies used for both the synthesis of the individual components and their integration into multifunctional hybrid systems, including wet-chemistry, coprecipitation, gelation, mixing, electrospinning and freeze-drying, are inherently compatible with sustainable manufacturing principles. These techniques typically rely on low-toxicity reagents, mild reaction conditions, and relatively low processing temperatures, thereby reducing energy requirements and minimizing the generation of hazardous by-products. In addition to their environmental benefits, such approaches facilitate the development of safe, scalable, and environmentally responsible multifunctional and multifaceted platforms.

### 7.2. Crosslinking-Ionic Gelation Synergy (BDDE and KCl)

The use of the FDA-approved crosslinking agent 1,4-butanediol diglycidyl ether (BDDE) to covalently bind the amino and hydroxyl groups of CS and κ-C significantly enhanced the structural integrity and stability of the resulting sponges and fibers. Furthermore, subsequent potassium chloride (KCl)-mediated ionic reticulation induced the aggregation of κ-C double helices, providing additional structural reinforcement and markedly improving the moldability, durability, and mechanical robustness of the system. This synergistic combination of covalent crosslinking and ionic gelation resulted in mechanically stable constructs capable of maintaining their structural and functional performance under a variety of physiological conditions, thereby supporting prolonged therapeutic actions. Importantly, this dual-stabilization strategy enabled precise modulation of both the mechanical properties and degradation kinetics. In the case of the 3D sponges, the resulting materials exhibited high resilience and the ability to fully recover their shape following mechanical compression under wet conditions, demonstrating their suitability for use in dynamic biological environments. Similarly, in electrospun nanofibrous systems, the mechanical properties, including Young’s modulus, tensile strength, and strain at failure, were finely tuned by varying the exposure time to basic ionic gelation at 50 °C. As a result, various nanofiber compositions displayed substantially enhanced elasticity, structural stability, and resistance to degradation, further broadening their potential for biomedical and tissue-engineering applications.

### 7.3. Structural, Textural and Porous Strategy

The use of CS or CS-based blends enabled the fabrication of highly porous structures with a broad pore-size distribution, a characteristic that is particularly advantageous for tissue engineering and wound-healing applications. Notably, the addition of κ-C and CS into polycaprolactone (PCL) formulations to produce electrospun nanofibers not only improved solution spinnability but also significantly enhanced storage stability, allowing the precursor solution to be preserved at −20 °C for up to 30 days without compromising fiber morphology or quality. This represented a substantial improvement over conventional PCL-based formulations, which typically exhibit limited storage stability and undergo degradation within a relatively short period. In the case of the 3D hybrid sponges, the integration of engineered SBA-15@Fe_3_O_4_ composites within these marine polysaccharide matrices generated highly interconnected architectures exhibiting hierarchical porosity across multiple length scales, including the coexistence of macro-, meso-, and microporous domains. The polysaccharide matrix provided macropores (≥10 μm), which were essential for cell infiltration, migration, vascularization, and tissue ingrowth. Simultaneously, the embedded SBA-15 composite, characterized by open cylindrical mesopores between 8−15 nm, markedly increased the specific surface area and surface roughness of the sponges, significantly enhancing surface topography for cell adhesion and colonization.

### 7.4. Synergistic Biological Responses

**Sustained and spatiotemporal drug release:** The highly ordered mesoporous structure of SBA-15 acted as an efficient drug reservoir, enabling the encapsulation and protection of sensitive therapeutic molecules, such as simvastatin, against premature degradation. When incorporated into hybrid systems, SBA-15 provided high drug-loading capacity and generated a biphasic first-order release profile. This release pattern consisted of an initial burst release, facilitating the rapid attainment of therapeutic levels, followed by a sustained diffusion-controlled release phase that prolonged drug availability for periods exceeding 30 days (up to 1400 h). Such long-term release kinetics are particularly advantageous for chronic wound-healing applications, where continuous therapeutic stimulation is often required to modulate inflammation, promote tissue regeneration, and minimize the frequency of dressing replacement.

**Biocompatibility and bioactivity:** The developed materials supported the adhesion and proliferation of a variety of cell types, including the bone marrow-derived stromal cell line ST2, the human osteosarcoma cell line MG-63, MC3T3-E1 preosteoblasts, and RAW 264.7 macrophages, demonstrating their broad cytocompatibility and suitability for regenerative applications. Furthermore, the dual incorporation of SBA-15@Fe_3_O_4_ and nano-hydroxyapatite (nHAp) into some sponges’ formulation promoted the rapid formation of bone-like apatite layers following 7–14 days of immersion in simulated body fluid (SBF), confirming their high bioactive behaviour. While nHAp contributed significantly to osteoconductivity and biomineralization, the engineered SBA-15@Fe_3_O_4_ composites imparted magnetic responsiveness and increased surface roughness, enhancing protein adsorption, cell adhesion, and cellular anchorage. The synergistic combination of these components created a favorable microenvironment for cell-material interactions, ultimately supporting tissue regeneration.

Similarly, the electrospun nanofibrous systems exhibited pronounced bioactive behavior when exposed to different physiological media, as evidenced by the formation of nanoparticulate deposits on their surfaces. This biomimetic response further highlights their potential to promote cellular attachment, proliferation, and regenerative processes in biomedical applications.

**Multifunctional theranostic capabilities:** The use of engineered SBA-15@Fe_3_O_4_ composites, in which SPIONs were covalently attached to SBA-15, enhanced the magnetic responsiveness of the biopolymer sponges, enabling remote manipulation under external magnetic fields. These magnetic nanocomposites also generated efficient localized heating during magnetic stimulation, achieving SAR values of up to 22.44 Wg^−1^ while minimizing thermal damage to adjacent tissues. The resulting thermal response could be tuned from mild hyperthermia to targeted cell ablation, depending on the application requirements. Moreover, the integration of magnetic hyperthermia and imaging capabilities within a single system underscores the theranostic potential of these multifunctional platforms.

### 7.5. Potential Synergistic Effects of the Proposed Multifunctional System in the Diabetic Wound Microenvironment

**Influence of the diabetic wound pH on system performance:** Diabetic wounds often exhibit a mildly acidic microenvironment (pH~5.5–6.5) as a consequence of bacterial infection, inflammation, and limited oxygen supply, all of which contribute to delayed healing. pH-responsive chitosan dressings are designed to exploit these pathological conditions by enabling on-demand release of therapeutic agents, including antimicrobial compounds and pro-regenerative molecules [177]. This responsiveness arises from the ionizable amino groups (–NH_2_) within the chitosan backbone, whose protonation state varies with fluctuations in local pH. Consequently, chitosan solubility is governed by protonation of its free amine groups (pKa ≈ 6.3–6.5). Below pH ~6.5, these amine groups become protonated, producing a positively charged, water-soluble polymer, whereas at neutral-to-basic pH chitosan precipitates due to loss of this charge-driven solubility [40]. These reversible interactions enable the hydrogel to swell, contract, or degrade as needed. The extent of this response is further influenced by factors such as the degree of deacetylation, molecular weight, and crosslinking strategy. These pH-dependent structural changes enable precise drug release, localized antibacterial action, and restoration of a favorable wound environment that supports tissue repair [178]. Meanwhile, mesoporous silica nanoparticles often exhibit accelerated cargo release under acidic conditions due to enhanced silica network hydrolysis and altered electrostatic interactions. The acidic pH effect on MSNs is of significant importance for their application in drug delivery [179]. Such pH-responsive drug delivery system can enhance wound-healing outcomes by promoting localized and on-demand release of therapeutic agents, thus improving treatment efficiency while minimizing systemic exposure and potential side effects [180]. Moreover, surface silanol groups on MSNs can reversibly coordinate divalent cations, thereby modulating release kinetics through adsorption-desorption equilibria and contributing to sustained therapeutic delivery. This effect may be further enhanced under mildly acidic conditions, where therapeutic ions such as Cu^2+^ and Zn^2+^ can be released more readily from ion-containing phases. In contrast, SPIONs generally remain stable in mildly acidic diabetic wound environments (pH ~5.5–6.5), particularly when protected by polymeric or inorganic surface coatings [181]. Significant dissolution of the iron oxide core and release of Fe^2+^/Fe^3+^ ions typically occur under more strongly acidic conditions (pH < 4–5). Therefore, at the pH range characteristic of diabetic wounds, SPIONs are generally expected to maintain their magnetic properties and structural integrity while remaining biologically functional.

**Synergistic interactions between therapeutic ions and MSNs:** MSNs are widely recognized for their exceptional capacity to load a broad range of therapeutic agents. Therefore, interactions occurring between therapeutic ions and mesoporous silica surfaces may favor ion retention and loading within the mesoporous system. When comparing the pore size of MSNs, which typically fall within the mesoporous range (2–50 nm), with the size of therapeutic metal ions such as Cu^2+^ and Zn^2+^, which are several orders of magnitude smaller (approximately 0.3–2 Å), pore blockage by these ions is not expected. Based on our knowledge and unpublished data currently under review on multi-ion-loaded SBA-15 platforms for ionic medicine applications in bone and dental tissue engineering, nitrogen sorption analyses have demonstrated that the incorporation of multiple therapeutic ions can lead to a moderate decrease in pore size and pore volume. However, these changes do not significantly alter the original mesostructural characteristics of SBA-15. These findings indicate that ion loading does not substantially alter the original pore architecture, although it may influence both the transport and release profile of ions. Accordingly, interactions between therapeutic ions and surface silanol groups may enhance ion retention and modulate release kinetics, promoting a sustained release that could ultimately improve the biological performance of ion-containing systems in the diabetic wound microenvironment. Similar behavior was reported by Vitázková, et al. [182] who demonstrated that Co- and B-co-doped mesoporous bioactive glass (MBG) nanoparticles exhibited the highest degradation and therapeutic ion release rates owing to their larger specific surface area, which promoted solid-liquid interfacial interactions and ion-exchange processes. In contrast, the corresponding SiO_2_-coated particles displayed a more regulated release behavior, prolonging the delivery of therapeutic ions while maintaining comparable Si release. These findings suggest that the silica shell containing silanol groups acts as a selective diffusion-regulating barrier, modulating ion transport and release kinetics, which contributed to improved biological performance.

**Synergistic interactions between therapeutic ions and SPIONs:** The presence of SPIONs may enable magnetic field-dependent modulation of ion diffusion through the chitosan matrix, particularly under magnetic hyperthermia conditions. By tuning the magnetic field strength and frequency, localized heating can be generated, which may enhance ion mobility and diffusion. The extent of this effect depends on the specific application (e.g., wound healing, antibacterial activity, or tissue engineering), as well as the composition and coating of the magnetic nanoparticles. Iron oxide-based nanoparticles are generally preferred for in vivo magnetic hyperthermia because of their strong magnetic response (M^bulk satd,^ ≈ 80–90 emu g^−1^), biocompatibility, and low toxicity [147]. Based on our current knowledge, no significant effect on ion diffusion has been observed under conventional tissue engineering conditions, however, an influence through magnetic hyperthermia cannot be excluded and requires further investigation.

In this context, the further incorporation of therapeutic ions into this type of hybrid formulations, either through controlled complexation with chitosan or in the form of metallic nanoparticles, represents a promising strategy for enhancing and broadening their therapeutic potential. Such integration can generate synergistic effects by combining the characteristics of therapeutic ions with the unique biological and physicochemical properties of chitosan, MSNs, and SPIONs. Collectively, these interactions may impart enhanced antibacterial and anti-inflammatory functionality while promoting cell proliferation, angiogenesis, extracellular matrix deposition, and tissue regeneration. As a result, these multifunctional platforms hold considerable promise for accelerating wound healing and improving tissue repair outcomes.

## 8. Critical Comparison of the Proposed Multifunctional Platform with State-of-the-Art Dressings for Diabetic Wound Healing

Over the past five years, considerable research efforts have focused on the development of multifunctional platforms for diabetic wound healing through the integration of diverse bioactive components. Most reported systems are restricted to relatively simple two- or three-component architectures, typically consisting of natural polymer matrices, such as chitosan, hyaluronic acid, or collagen, combined with a single bioactive cargo, including therapeutic agents, growth factors, or nanoparticle systems. According to the current state-of-the-art, naturally derived polymers are generally favored over synthetic counterparts owing to their intrinsic biocompatibility, biodegradability, and bioactivity, all of which are highly beneficial for tissue repair and regeneration. Among them, hyaluronic acid and collagen have demonstrated remarkable potential for promoting angiogenesis, cell proliferation, and extracellular matrix remodeling. Nevertheless, their widespread use remains limited by high production and purification costs, as well as challenges associated with large-scale manufacturing and availability, hindering their regular use in cost-effective wound care products, especially in resource constrained environments. In contrast, chitosan is derived from the abundant and inexpensive natural resource chitin, commonly obtained from crustacean shell waste, offering comparable biocompatibility while providing substantially greater economic feasibility and manufacturing scalability and accessibility. These advantages position chitosan as an attractive matrix for the development of cost-effective advanced wound dressings. Furthermore, as summarized in Table 5, most currently available multifunctional dressings rely on relatively simpler compositions, highlighting the opportunity to develop more integrated platforms capable of simultaneously addressing the multiple pathological features of diabetic wounds.

**Table 5:** State of the art multifunctional dressings reported for diabetic wound healing.

System & Polymer Matrix	Functional Payload	Release/Crosslinking Mechanism	Key Reported Outcome	Limitations	Ref
Carboxymethyl chitosan (CMCS) + oxidized hyaluronic acid	Curcumin-loaded mesoporous polydopamine NPs	Dynamic imine bonds + electrostatic interaction	Dual-stage release (rapid + sustained); strong antioxidant, hemostatic, antibacterial activity	No embedded diagnostic/tracking function; long-term in vivo stability of dual-drug release not established	[183]
PEG-co-poly(glycerol sebacic acid)–chitosan (PC)	Metformin + graphene oxide	Dual dynamic bonds (Schiff base + phenyl boronate ester)	pH/glucose dual-responsive release; extensively characterized (NMR, FTIR, SEM, rheology, in vivo hemostasis)	Complex multi-step synthesis (dual dynamic bonding chemistry); no diagnostic capability; GO long-term biocompatibility not addressed	[184]
Aminated gelatin + oxidized dextran	Zinc oxide nanoparticles	Dynamic Schiff base	Inflammation-responsive sequential release (hemostatic → antibacterial → angiogenic)	Single nanomaterial type limits functional versatility; no imaging/monitoring component; ZnO dose-dependent cytotoxicity not discussed	[185]
Hyaluronic acid-ADH + oxidized dextran	Laponite nanoplatelets + LL-37 mimetic peptide (W379)	Reversible Schiff base	Broad-spectrum antibacterial, biofilm inhibition, re-epithelialization	Peptide-based systems face stability/cost/scale-up challenges; no diagnostic or tracking function; no ion-release component	[186]
Bacterial cellulose	Copper nanoparticles + polydopamine	Self-polymerization	Skin-comparable conductivity; dual function as dressing + motion/microexpression sensor	Sensing function is electrical/motion-based only, not diagnostic imaging; Cu-NP long-term systemic safety not addressed; bacterial cellulose production scalability is a known bottleneck	[187]
ZIF-8 (Zn-based MOF) + bacterial cellulose sponge	Antioxidant naringin	Hydrogen bonding (ZIF-8–BC hydroxyls)	High exudate absorption, enhanced mechanical strength, low density	No stimuli-responsive release mechanism reported; no diagnostic function; MOF (ZIF-8) long-term degradation/toxicity profile not established	[188]

### 8.1. Advantages and Disadvantages of the Proposed Multifunctional System

Compared with the systems outlined in Table 2 and Table 5, the proposed platform provides notable practical advantages in the preparation of its inorganic components. Both MSNs and SPIONs can be produced through simple, scalable, and economically viable synthesis routes, such as sol-gel, co-precipitation, and wet-chemistry methods. These well-established fabrication routes facilitate large-scale production while maintaining precise control over particle size, morphology, and physicochemical properties [189]. These processes are conducted in aqueous media under simple and readily controllable conditions, typically at moderate temperatures (<100 °C). Moreover, they represent environmentally benign synthesis routes, employing water, acetic acid, or other aqueous media as the primary solvent system, thereby avoiding the use of organic solvents, cytotoxic crosslinking agents, and additional processing steps such as UV-initiated crosslinking [190]. This straightforward fabrication strategy is complemented by a broader and more mechanistically diverse spectrum of therapeutic functions, including angiogenic, antibacterial, antioxidant, and regenerative activities, thereby offering greater functional complexity than the single-agent or single-nanoparticle systems commonly described in current research. While the four-component design expands the simultaneous physicochemical and biological functionalities compared with currently reported dressings, it also introduces compatibility, stability, and scale-up challenges that have not yet been systematically resolved in the literature, highlighting important areas for future development and optimization.

The principal limitation of the proposed multifunctional platform stems from its multi-component composition. First, it introduces a higher degree of component-component interactions, including chitosan-ion complexation and potential nanoparticle aggregation, which are generally absent in systems containing a single nanomaterial. Second, the incorporation of four independent components increases manufacturing complexity and may contribute to greater batch-to-batch variability. Third, regulatory approval and comprehensive characterization become more demanding, as the safety, stability, and efficacy of both the individual components and the integrated system must be rigorously evaluated. Consequently, the translation of such multifunctional platforms into clinical practice is likely to be more challenging than that of established two-component wound dressing systems.

### 8.2. Regulatory, Manufacturing, and Clinical Translation Challenges

Despite the promising therapeutic potential of chitosan-based multifunctional platforms that integrate MSNs, therapeutic ions, and SPIONs, several translational challenges must be addressed before clinical implementation.

**Regulatory considerations for combination products:** The proposed four-component system is likely to be classified as a combination product because it integrates multiple independently regulated constituents within a single therapeutic platform. As a result, regulatory approval would require comprehensive evaluation of the safety, efficacy, and manufacturing quality of each component, as well as validation of the overall risk-benefit profile of the integrated system. Consequently, the regulatory pathway is expected to be more complex than that of conventional single-component therapies, necessitating extensive characterization of component interactions, batch-to-batch reproducibility, and long-term safety and performance [191].

**Manufacturing and scalability challenges:** Chitosan is one of the most abundant naturally occurring polysaccharides and is widely recognized for its biodegradability and environmental sustainability. Nevertheless, large-scale production remains challenging because the properties of chitosan-based dressings are strongly influenced by the molecular weight and degree of deacetylation of the polymer, which can contribute to batch-to-batch variability. Similar challenges apply to inorganic nanoparticle components, as they are commonly produced through bottom-up synthetic approaches that often limit production scale and reproducibility. However, the industrial-scale manufacture of magnetic nanoparticles is already well established, with several companies (Micromod Partikeltechnologie GmbH, Chemicell, and Adámas Nanotechnologies) producing SPIONs at commercial volumes. These developments suggest that large-scale production of the proposed inorganic components is technically feasible, although further optimization will be required to ensure manufacturing consistency and regulatory compliance.

**Stability and shelf-life considerations:** Available evidence indicates that these materials exhibit favorable long-term stability under appropriately controlled storage conditions, including dry storage, protection from light, and regulated temperature and humidity. Nevertheless, successful clinical translation and commercialization will require the development of optimized packaging, preservation, and lyophilization strategies, together with standardized stability-testing protocols, to ensure the maintenance of material integrity, functionality, and shelf life throughout storage, transportation, and clinical application.

**Cost-effectiveness and health economic considerations:** The polysaccharide and inorganic components of the proposed platform are relatively inexpensive and environmentally sustainable compared with many existing wound-healing therapies. In addition, their fabrication relies on mild processing conditions and generally avoids high-temperature treatments, toxic organic solvents, and energy-intensive manufacturing steps, thereby supporting cost-effective, scalable, and environmentally responsible production [192].

**Immunogenicity and long-term safety:** To the best of our knowledge, preliminary in vivo studies evaluating chitosan sponges incorporating SBA-15 mesoporous silica and nanohydroxyapatite demonstrated promising outcomes in male rabbit models. Subsequent formulations further incorporating SPIONs through engineered SBA-15@Fe_3_O_4_ composites exhibited improved biological performance when evaluated in direct-contact assays using MC3T3 cells, with the previously in vivo-tested sponge serving as the control. Specifically, the magnetic composite-containing sponges promoted higher cell viability, adhesion, and colonization, highlighting the beneficial contribution of SPION-functionalized SBA-15 systems to the overall biological response [176]. These findings support the feasibility of further long-term safety and efficacy studies aimed at clinical translation. Nevertheless, the long-term safety, biodistribution, and clearance of advanced multifunctional therapeutic platforms remain insufficiently understood and warrant further investigation.

## 9. Conclusions and Future Perspective

Diabetic wounds remain one of the most challenging complications of diabetes to manage, owing to the combined burden of infection, hypoxia, impaired angiogenesis, oxidative stress, and persistent inflammation that characterize their pathological microenvironment. Conventional therapies and single-function wound dressings often provide only partial and predominantly passive solutions to this multifactorial condition. In this context, chitosan has emerged as an attractive biomaterial due to its intrinsic biocompatibility, biodegradability, hemostatic activity, antimicrobial properties, and versatile chemical structure, which can be tailored to create advanced therapeutic dressings that extend far beyond the role of a conventional wound covering. Nevertheless, despite the substantial progress achieved in the field, the current evidence base is predominantly limited to two-component chitosan-containing systems. The synergistic integration of chitosan-based matrices, therapeutic ions, mesoporous silica nanocarriers, and superparamagnetic iron oxide nanoparticles represents a promising strategy for the development of next-generation engineered platforms for diabetic wound management. Such multifaceted systems have the potential to combine stimuli-responsive and sustained therapeutic delivery with antimicrobial, angiogenic, antioxidant, and regenerative functionalities, while simultaneously enabling magnetic-field-assisted imaging, hyperthermia, targeting, and therapeutic modulation. The complementary interplay among these components may facilitate infection control, angiogenesis, cell migration, extracellular matrix formation, and tissue regeneration, thereby addressing multiple pathological aspects of diabetic wounds within a single platform. Although a fully integrated four-component system has not yet been reported, the growing body of evidence from related multifunctional platforms strongly supports the feasibility and therapeutic promise of this approach.

Despite its considerable potential, several challenges must be addressed before clinical translation can be achieved. The increased complexity of multi-component systems raises concerns related to component compatibility, physicochemical stability, manufacturing reproducibility, and scalable production. In addition, the interactions among therapeutic ions, chitosan, mesoporous silica, and magnetic nanoparticles remain insufficiently understood, particularly regarding release behavior, long-term stability, and synergistic biological effects. Safety and regulatory considerations also require careful attention. Although localized delivery is expected to reduce systemic exposure, the long-term biodistribution, clearance, accumulation, and potential toxicity of released metal ions and inorganic nanomaterials remain to be fully elucidated. Consequently, future studies should extend beyond local biocompatibility and include comprehensive assessments of systemic safety, organ distribution, immune responses, degradation products, and metal homeostasis. Moreover, the integration of multiple functional components is likely to increase regulatory complexity, requiring rigorous validation of safety, efficacy, manufacturing consistency, and long-term performance. Future research should focus on quantitatively evaluating ion release, cargo delivery, biological performance, and therapeutic efficacy under physiologically relevant diabetic wound conditions. Establishing clear relationships between composition, release kinetics, and biological outcomes, while benchmarking the integrated platform against its individual components, will be essential for elucidating potential synergistic effects. Ultimately, comprehensive preclinical and clinical studies will be required to determine the translational potential of fully integrated four-component theranostic platforms for diabetic wound management.

## Data Availability

Dataset available on request from the authors.
